# Cyclic compressive loading induces a mature meniscal cell phenotype in mesenchymal stem cells with an atelocollagen-based scaffold

**DOI:** 10.3389/fbioe.2024.1394093

**Published:** 2024-05-20

**Authors:** Shohei Oyama, Takashi Kanamoto, Kosuke Ebina, Yuki Etani, Makoto Hirao, Atsushi Goshima, Shunya Otani, Minami Hikida, Satoshi Yamakawa, Shohei Ito, Seiji Okada, Ken Nakata

**Affiliations:** ^1^ Department of Musculoskeletal Regenerative Medicine, Osaka University Graduate School of Medicine, Osaka, Japan; ^2^ Taisho Pharmaceutical Co., Ltd., Saitama, Japan; ^3^ Department of Medicine for Sports and Performing Arts, Osaka University Graduate School of Medicine, Osaka, Japan; ^4^ Department of Orthopaedic Surgery, Osaka University Graduate School of Medicine, Osaka, Japan; ^5^ Department of Orthopaedic Surgery, National Hospital Organization, Osaka Minami Medical Center, Osaka, Japan; ^6^ Department of Orthopaedic Surgery, Osaka Rosai Hospital, Osaka, Japan; ^7^ Department of Sports Medical Biomechanics, Osaka University Graduate School of Medicine, Osaka, Japan

**Keywords:** cyclic compressive loading, scaffold, three-dimensional culture, meniscus, mesenchymal stem cells

## Abstract

**Introduction:** Biomechanical stimulation is reportedly pivotal in meniscal regeneration, although its effect on mesenchymal stem cell (MSC) meniscal differentiation remains elusive. In this study, we investigated how cyclic compressive loading (CCL) could impact MSCs using three-dimensional cultures in atelocollagen-based meniscal substitute (ACMS).

**Methods:** We extracted MSCs from the meniscus, synovium, and articular cartilage, cultured them in three-dimensional cultures, and exposed them to CCL for 7 days. We then compared the transcriptomes of MSCs treated with and without CCL.

**Results:** Our RNA-seq analysis revealed that CCL induced significant transcriptome changes, significantly affecting chondrocyte-related genes, including SOX9, TGFB1, and PRG4 upregulation. CCL induced transcriptional differentiation of meniscus progenitors toward mature meniscal cells.

**Conclusion:** This study unveils the potential of mechanical stress in promoting MSC meniscal differentiation within ACMS. Our investigations provide new insights for mechanisms underlying meniscal regeneration with ACMS.

## Introduction

The meniscus is a crescent-shaped fibrocartilaginous structure between the femoral condyles and tibial plateau cartilage (Fox et al., 2015). It serves as a shock absorber, distributing loads and providing a low-friction surface for joint loading and movement, essential for maintaining normal knee biomechanical function. The outer, vascularized region features a fibrous matrix dominated by type I collagen, while the inner region of the meniscus, avascular and less capable of self-repair, primarily consists of a cartilaginous matrix rich in type II collagen and chondrocyte-like cells (Makris et al., 2011; Zhou et al., 2022). Meniscal damage is associated with degenerative changes in the knee joint ultimately leading to osteoarthritis (OA) (Berthiaume, 2005; Englund et al., 2009; Badlani et al., 2013). Due to the future risk of OA, current orthopedic practice prioritizes preserving meniscus integrity using various surgical techniques (e.g., defect closure with sutures and allograft transplantation) (Johnson et al., 1999; Lee et al., 2019). However, such current approaches pose challenges in long-term functional repair for patients with complex meniscal injuries or defects.

Tissue engineering approaches have incorporated biomaterial scaffolds as a strategy for repairing meniscal injuries with defects (Scotti et al., 2013; Mauck and Burdick, 2015). Such scaffolds comprise various synthetic or natural materials (e.g., collagen, alginate, polylactides, polyglycolides, and silk) (Makris et al., 2011). Certain meniscal scaffolds are currently applied in clinical practice for meniscus repair. However, using such scaffolds holds challenges, leading to allergic reactions and incomplete morphological or functional regenerative capacity (van Tienen et al., 2009; Bulgheroni et al., 2016; Ranmuthu et al., 2019). To overcome these limitations, we developed a novel, bovine dermis-derived cross-linked type I atelocollagen-based meniscal substitute (ACMS). Atelocollagen is a form of collagen with the immunogenic telopeptides removed, ensuring compatibility and reduced immune response. Our ACMS exhibits mechanical properties comparable to the native meniscus, appropriate porosity for cell infiltration, and low immunogenicity (Shimomura et al., 2014; Hsieh and Srinivasan, 2020; Kanamoto et al., 2021). ACMS was transplanted into the meniscal defect lesion in a minipig model of a partial meniscal defect, leading to cell migration, scaffold resorption, and eventual meniscal regeneration with intrinsic extracellular matrix production over 9 months (Yokoi et al., 2019). For scaffold-based tissue regeneration cell sources, a growing consensus supports endogenous mesenchymal stem/progenitor cells (MSCs) as promising candidates. MSCs are present in various tissues (e.g., the meniscus, synovium, and articular cartilage) (Segawa et al., 2009; Muhammad et al., 2014; Seol et al., 2017) and their enhanced recruitment reportedly promoted cartilage repair using a type I collagen gel scaffold (Kubo et al., 2007). Moreover, endogenous meniscal cells and MSCs both infiltrated meniscus-derived scaffolds and repaired meniscus defects *in vitro* (Ruprecht et al., 2019). Furthermore, three-dimensional printed scaffolds with CTGF and TGF-β3 recruited endogenous stem and progenitor cells, promoting meniscal regeneration in a sheep model of knee injury (Lee et al., 2014). MSCs thus exhibit promising potential in tissue regeneration.

Biomechanical stimulation is crucial for meniscal development (McNulty and Guilak, 2015). Numerous studies have investigated the effects of mechanical loading on human meniscal cells, demonstrating that it can either enhance chondrogenic differentiation or exert no significant change (Suzuki et al., 2006; Zellner et al., 2015; Szojka et al., 2021). Furthermore, mechanical stimulation has been reported to influence the meniscal differentiation of MSCs in several studies. Mechanical stimulation reportedly causes increased chondrogenic marker gene expression (e.g., type II collagen, aggrecan, TGF-β1, and SOX9) in MSC-related chondrogenesis (Fahy et al., 2018). Cyclic compression of collagen meniscus implants seeded with human MSCs increased procollagen type I and III peptide production (Petri et al., 2012). Moreover, rabbit MSCs displayed upregulated fibrogenic genes (e.g., COL1A1, FN1, and TNC) combined with compressive strain and growth factors (Zhang et al., 2019b). While previous studies have indicated that biomechanical stimuli promote MSC differentiation toward meniscal cells, meniscus-specific markers were not utilized for evaluation. Recently, advances in RNA-seq analysis of human meniscal tissue have led to the identification of unique markers characteristic of meniscal cells (Sun et al., 2019). These findings pave the way for a more accurate understanding of the differentiation in meniscal cells.

In this study, we aimed to investigate how cyclic compressive loading (CCL) stimulation affects meniscal differentiation of MSC derived from the meniscus, synovium, and articular cartilage combined with transcriptome analysis and meniscus-specific gene markers.

## Methods

### Ethics statement

This study was conducted using cells derived from human meniscus, synovium, and articular cartilage and was approved by the Osaka University Institutional Ethical Committee (approval ID 16085-4). Written informed consent was obtained from all participants and all methods were performed in accordance with the relevant guidelines and regulations.

### Cell isolation and culture

Human meniscus, synovium, and articular cartilage were obtained from a total of 9 patients (mean age, 71.0 years; range, 61–80 years; 3 men and 6 women) with knee OA or osteonecrosis who underwent total knee arthroplasty within 48 h after surgery. All tissues were rinsed with phosphate-buffered saline (PBS), minced, and digested with 0.2% (w/v) type IV collagenase (C5138-1G; Sigma-Aldrich, St. Louis, MO) in PBS with 1% penicillin/streptomycin (Thermo Fisher Scientific, Waltham, MA) for 3–4 h at 37°C. After centrifugation, the cells were resuspended in Dulbecco’s Modified Eagle Medium/Nutrient Mixture F-12 Ham (DMEM/F12; Sigma-Aldrich) supplemented with 20% fetal bovine serum (FBS; 175012-500ML; Nichirei, Tokyo, Japan) and 1% penicillin/streptomycin, then passed through a 100-μm nylon filter (Corning, Corning, NY) to remove debris. Meniscus-, synovium-, and articular cartilage-derived cells (MCs, SCs, and ACs, respectively) were seeded onto 150-mm diameter plastic culture dishes, and cultured in a monolayer at 37°C in 5% CO_2_ atmosphere. The medium was changed once a week. After 10–14 days of primary culture, the subconfluent cells were washed with PBS, harvested by treatment with trypsin-EDTA (0.25% trypsin and 1 mM EDTA; Thermo Fisher Scientific), and replated from 1:2- to a 1:3-diluted DMEM/F12 supplemented with 10% FBS and 1% penicillin/streptomycin. Cell passages were continued similarly with media changes twice a week. At passages 3–6, the cells were used in the following experiments.

### Flow cytometry

MCs, SCs, and ACs were suspended in 100 μL of PBS, containing 0.5% (w/v) bovine serum albumin (Nakalai Tesque, Kyoto, Japan) and 5 μL of a fluorescence-coupled antibody at room temperature for 1 h in the dark. For the fluorescence-coupled antibodies, PE anti-human CD73 (#344003), APC anti-human CD90 (#328113), FITC anti-human CD105 (#323203), FITC anti-human CD45 (#368507), and APC anti-human CD34 (#343509) were purchased from BioLegend (San Diego, CA). For the isotype control, APC mouse IgG1 κ isotype ctrl antibody (#400120), FITC mouse IgG1 κ isotype ctrl antibody (#400108), and PE mouse IgG1 κ isotype ctrl antibody (#400112) were also purchased from BioLegend. The cells were then washed twice, centrifuged, and submitted to FACS analysis. Cell fluorescence was evaluated using a BD FACSVerse instrument (Becton Dickinson, Franklin Lakes, NJ) and the data were analyzed using the BD FACSuite Software Application.

### Multi-differentiation potential evaluation

The multi-differentiation potential of MCs, SCs, and ACs was evaluated for chondrogenesis, osteogenesis, and adipogenesis. The cells were seeded onto plastic surfaces in 24-well plastic plates at a density of 2.0 × 10^4^ cells/well in DMEM/F12 supplemented with 10% FBS and 1% penicillin/streptomycin for 3 days. For chondrogenesis, the cells were cultured in DMEM/F12 supplemented with 1% FBS, 1% penicillin/streptomycin, 1% ITS (insulin-transferrin-selenium; Corning), 1% MEM non-essential amino acid (Fujifilm Wako Pure Chemical Co. Ltd., Osaka, Japan), 10 ng/mL human TGF-β1 (100-21-10μg; PeproTech, Rocky Hill, NJ), 10 ng/mL BMP2 (Osteo Pharma, Osaka, Japan), and 50 μg/mL ascorbic acid (Sigma-Aldrich). For osteogenesis, the cells were cultured in Stem pro osteogenesis differentiation kit (Thermo Fisher Scientific), and for adipogenesis, cells were cultured in Stem pro adipogenesis differentiation kit (Thermo Fisher Scientific). Media were replaced every three to 4 days. After 14 days of differentiation induction, chondrogenesis, osteogenesis, and adipogenesis were tested by stainings with Alcian Blue solution pH2.5 (Fujifilm Wako Pure Chemical Co. Ltd.), alkaline phosphatase (ALP; Promega, Madison, WI), and Oil Red O (Muto Pure Chemical Co. Ltd., Tokyo, Japan), respectively.

### Histology

The cell-scaffold constructs with or without CCL were fixed in fresh 4% paraformaldehyde (Wako Pure Chemicals) overnight at 4°C, immersed in 30% sucrose solution, then frozen in OCT compound (Sakura Finetek, Tokyo, Japan) at −80°C. The cryosections were performed at a thickness of 15 μm using Cryofilm (SECTION-LAB Co. Ltd., Hiroshima, Japan), washed with PBS, and stained with hematoxylin and eosin. The images were obtained using a BX53/DP74 microscope (Olympus, Tokyo, Japan).

### Actin cytoskeleton detection

For 2D experiments, MCs, SCs, and ACs were seeded on Nunc Lab-Tek II Chamber Slide System 8 well (2.0 × 10^4^ cells/well; Thermo Fisher Scientific). Subconfluent cells were washed in PBS, fixed with fresh 4% paraformaldehyde, then incubated with 0.5% Triton-X/PBS solution for membrane permeabilization. Immunohistochemistry was performed using Acti-stain 555 Fluorescent Phalloidin (PHDH1, Cytoskeleton Inc., Denver, CO) for 30 min at room temperature. The cell nuclei were stained with 4′6-diamidino-2-phenylindole (DAPI, Vector Laboratories, Inc., Burlingame, CA). For 3D, the cell-scaffold construct cryosections were prepared at the thickness of 10 μm, permeabilized, and blocked with Blocking One Histo (Nacalai Tesque) for 1 h at room temperature. Immunohistochemistry was performed with PHDH1 overnight at 4°C and the cell nuclei were stained with DAPI. The images were obtained using a DMi8 (Leica, Wetzlar, Germany) microscope.

### 3D culture in the ACMS scaffold

The cells were harvested and seeded on collagen scaffolds to produce 3D constructs as previously described (Muroi et al., 2007; Akamine et al., 2012; Shimomura et al., 2014). Briefly, the cultured cells (5 × 10^5^ cells/scaffold) were suspended in DMEM/F12 supplemented with 10% FBS and 1% penicillin/streptomycin, then gently mixed with an equal volume of 1% atelocollagen gel (Koken Co. Ltd., Tokyo, Japan) on ice to produce a cell suspension in 0.5% collagen solution. The cell suspension was incorporated into collagen scaffolds (Atelocollagen Sponge Mighty, Koken; 5 and 3 mm in diameter and thickness, respectively) by centrifugation at 500 *g* for 5 min. The collagen sponges displayed an interconnected pore size of 30–200 μm, were fabricated via freeze-drying of 10% collagen gel and cross-linking to reinforce the mechanical properties, and can withstand a compressive loading of up to 40 kPa (Muroi et al., 2007; Akamine et al., 2012; Shimomura et al., 2014; Kanamoto et al., 2021). The cell-scaffold constructs were then incubated at 37°C for gelation to produce 3D cell-scaffold constructs. The constructs were cultured on 96-well plates in 200 μL of DMEM/F12 supplemented with 10% FBS and 1% penicillin/streptomycin under free-swelling conditions at 37°C and in 5% CO_2_.

### CCL

**FIGURE 1:**
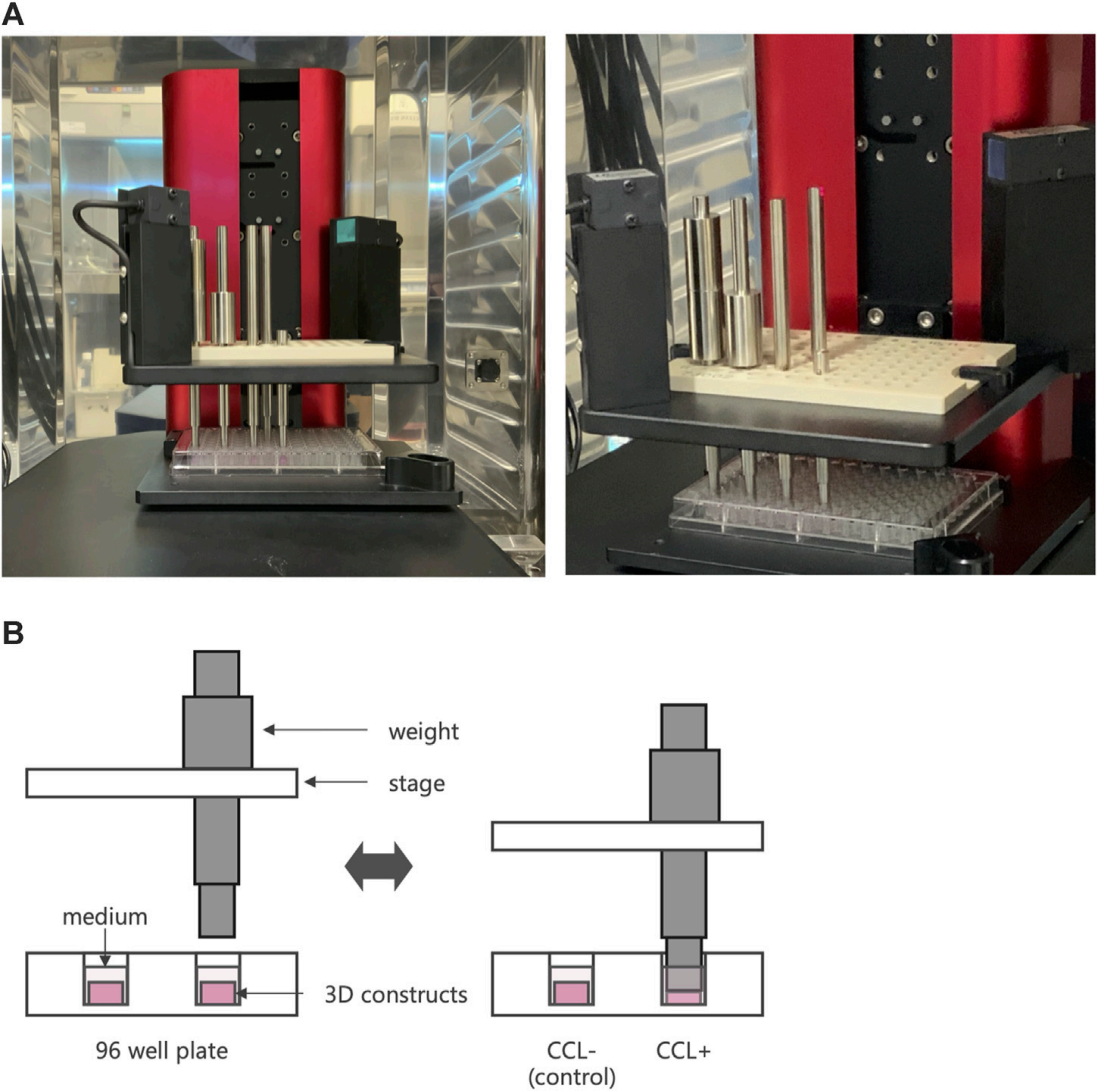
Cyclic compressive loading (CCL) on 3D constructs. **(A)** An image of a cyclic load bioreactor. The bioreactor was installed inside an incubator and operated at 37°C and 5% CO_2_ conditions. **(B)** Schematic representation of a bioreactor to apply CCL. A plate moves up and down to exert the load. When the piston touches the sample, it retracts from the plate, applying a consistent force.

After 7 days of incubation on 3D cell-scaffold constructs, the 3D constructs underwent CCL using a custom-designed apparatus, a cyclic load bioreactor (CLS7J; Technoview, Osaka, Japan), as previously described (Muroi et al., 2007; Akamine et al., 2012; Shimomura et al., 2014; Kanamoto et al., 2021) (Figures 1A, B). Briefly, using metal pistons, 40-kPa CCL was applied to the cell-scaffold constructs at 0.5 Hz for 1 h per day, followed by free-swelling for 23 h. This procedure was repeated seven times over a period of 7 days. The cell-scaffold constructs were collected 6 hours after the last CCL for the gene expression analysis and histological examination, and the cell-scaffold constructs and culture supernatant were collected 24 h after the last cyclic compressive loading for measurement of protein concentration, DNA content, and biomechanical properties.

### RNA extraction and quantitative real-time RT-PCR

The frozen cell-scaffolds were crushed and homogenized using a stainless beads shocker. Total RNA extraction and reverse transcription were performed using Trizol (Thermo Fisher Scientific), PureLink RNA Purification kit (Thermo Fisher Scientific), and High-Capacity RNA-to-cDNA kit (Thermo Fisher Scientific) according to their manufacturer’s instructions. Quantitative PCR was performed using Power SYBR Green Master Mix and QuantiStudio 7 Pro Real-Time PCR System (Thermo Fisher Scientific). Specific primers were used for target genes (Table 1). The expression of the target genes was normalized to that of the reference gene, hypoxanthine phosphoribosyltransferase 1 (HPRT1).

**TABLE 1:** RT-qPCR primer sequences.

Gene	Forward primer	Reverse primer
*HPRT1*	TGC​TCG​AGA​TGT​GAT​GAA​GG	TCC​CTG​TTG​ACT​GGT​CAT​T
*CNN1*	GCC​TCT​GTT​CTC​AGC​GTC​AGT	TCG​ATC​CAC​TCT​CTC​AGC​TCC
*MYLK*	GGG​GAC​TTT​CAG​CCT​TGT​GA	GAC​CAA​GCT​GCT​TCG​CAA​AA
*FOSL1*	GCC​TCT​GAC​CTA​CCC​TCA​GT	CAG​TTT​GTC​AGT​CTC​CGC​CT
*BMP2*	TCC​TGA​GCG​AGT​TCG​AGT​TG	TCT​CCG​GGT​TGT​TTT​CCC​AC

### RNA-seq analysis

Library preparation was performed using a TruSeq stranded mRNA sample prep kit (Illumina, San Diego, CA) according to the manufacturer’s instructions. Sequencing was performed on an Illumina NovaSeq 6,000 platform in a 100 bp paired-end mode. Sequenced reads were mapped to the human reference genome sequences (hg19) using TopHat v2.0.13 in combination with Bowtie2 ver. 2.2.3 and SAMtools ver. 0.1.19. The fragments per kilobase of exon per million mapped fragments (FPKMs) was calculated using Cufflinks version 2.2.1. We identified DEGs using R package DESeq2 version 1.34.0 comparing non-loading samples with loading samples. We use the default Wald test for differential expression analysis. The method used for adjusting *p*-values was Benjamini–Hochberg method. DEGs were defined as genes with adjusted *p*-value less than 0.05 and absolute value of fold change greater than 1. For functional enrichment analysis of GO categories and KEGG pathways (Kanehisa and Goto, 2000), we employed the software package ClusterProfiler version 4.2.2 in R. The results from the enrichment analysis were adjusted for multiple testing using the Benjamini–Hochberg method to control the false discovery rate (FDR). Both GO terms and KEGG pathways were considered significantly enriched if the adjusted *p*-value (FDR) was less than 0.01.

### Cell type deconvolution analysis

Relative cell type proportions were inferred using the marker-based decomposition method implemented in the BisqueRNA package (Jew et al., 2020) in R. The single-cell RNA-seq data reported by Sun et al. (Sun et al., 2019) were used as reference data and cell type marker genes were identified using the Seurat package (Hao et al., 2021).

### DNA content measurement

The cell-scaffold constructs were harvested 24 h after the last loading, and digested in 500 μL of papain/PBS (125 μg/mL papain; Sigma-Aldrich) overnight at 65°C (Ruprecht et al., 2019). After centrifugation at 12,000 × *g* for 5 minutes, supernatants were used to determine fluorometrically DNA concentration with Qubit 1X ds DNA HS Assay Kit (Thermo Fisher Scientific) and Qubit 4.0 Fluorometer (Thermo Fisher Scientific).

### Transforming Growth Factor β1 measurement

Homogeneous time-resolved fluorescence (HTRF^®^; Perkin Elmer, Waltham, MA) assay was performed to assess Transforming Growth Factor (TGF) -β1 concentration in culture supernatant. The culture supernatants were harvested 24 h after the last loading, centrifuged to precipitate insoluble aggregates. Human TGF-β1 concentration was determined using human and mouse TGF-β1 kit (Cisbio) according to the manufacturer’s protocol. The concentration of TGF-β1 was expressed as protein amounts per DNA amounts.

### Statistical analysis

For the experiments of flow cytometry, multi-differentiation potential evaluation, RNA-seq, RT-qPCR, and TGF-β1 measurement at least three independent experiments with three or six different donors were performed. For analyzing the results of the RT-qPCR, the statistical significance of differences between 2D group and 3D without CCL group and between 3D without CCL group and 3D with CCL group was determined by Wilcox rank sum test. For analyzing the results of the TGF-β1 measurement, the statistical significance of differences between 3D without CCL group and 3D with CCL group was determined by Welch’s *t*-test. A significance level of 95% with a *p*-value of 0.05 was used for all the statistical tests. R version 4.1.1. software was used for all the statistical analyses.

## Results

### Meniscus-, synovium-, and articular cartilage-derived cell characterization

**FIGURE 2:**
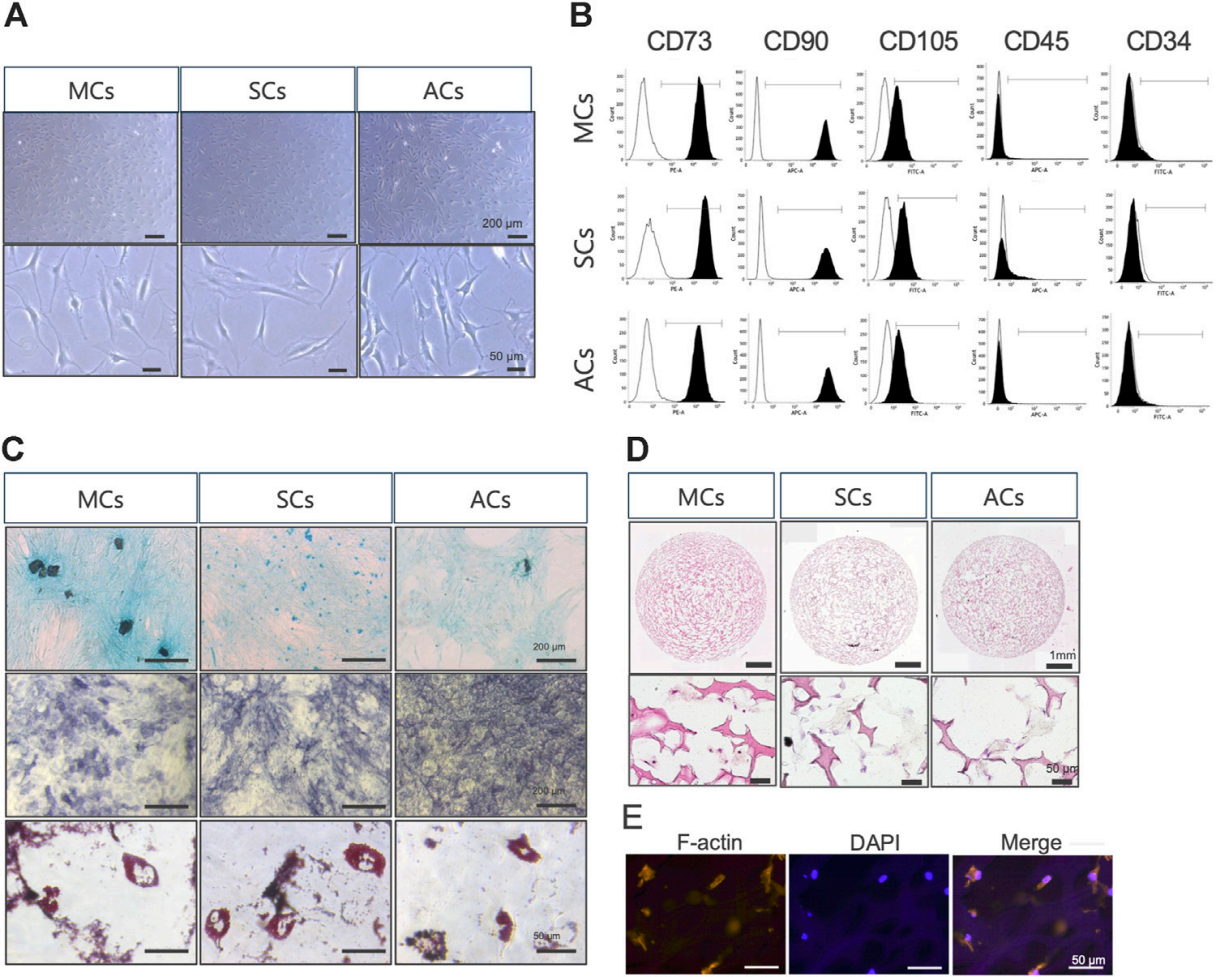
Meniscus-derived cells (MCs), synovium-derived cells (SCs), and articular cartilage-derived cells (ACs) properties as mesenchymal stem cells (MSCs) **(A)** Microscopic images of *in vitro* cultured MCs (left), SCs (center), and ACs (right). The top and bottom rows were imaged at low and high magnifications (scale bars: 200 and 50 μm), respectively. **(B)** Flow cytometry data illustrating surface CD marker expressions characteristic of MSCs in MCs (top), SCs (center), and ACs (bottom). The white and black areas indicate isotype controls and target marker expression, respectively. The range line demarcates the positive area for the target marker. Representative plots from each of the three biological replicates are displayed, demonstrating the consistency of the observed trends. **(C)** MCs (left), SCs (center), and ACs (right) on day 14 of differentiation, stained with Alcian Blue (upper), alkaline phosphatase (middle), and Oil Red O (lower). Representative examples from the three biological replicates. Black and white scale bars: 1 mm and 50 μm, respectively. **(D)** Hematoxylin and eosin staining images of MCs (left), SCs (center), and ACs (right) on day 14 of 3D culture. The top and bottom rows were imaged at low and high magnifications (scale bars: 1 mm and 50 μm), respectively. **(E)** Fluorescent immunohistochemical images of the MCs cytoskeleton on day 14 of 3D culture. The cytoskeleton and nuclei are stained with F-actin and DAPI, respectively. Scale bar: 50 μm.

First, we examined whether meniscus-, synovium-, and articular cartilage-derived cells (MCs, SCs, and ACs) exhibited MSC characteristics (Dominici et al., 2006). In plate cultures, all cell types adhered to the bottom of a plastic dish and formed a typical spindle-shaped fibroblast-like phenotype (Figure 2A). Our flow cytometry analysis revealed that all these cells expressed MSC (CD73, CD90, and CD105) but not hematopoietic (CD34 and CD45) markers (Figure 2B). All these cells could differentiate into chondrocytes, osteocytes, and adipocytes when cultured in chondrogenic, osteogenic, and adipogenic media, respectively (Figure 2C). Taken together, 2D-cultured MCs, SCs, and ACs exhibited MSC characteristics. Subsequently, we established three-dimensional (3D) MC, SC, and AC cultures. We performed HE- and immunostainings to examine the cells within the scaffold after 14 days of culture and observed viable cells within the scaffold complex, exhibiting a cartilage-like circular morphology (Figures 2D, E).

### Transcriptomic responses to CCL in 3D-Cultured MCs, SCs, and ACs

**FIGURE 3:**
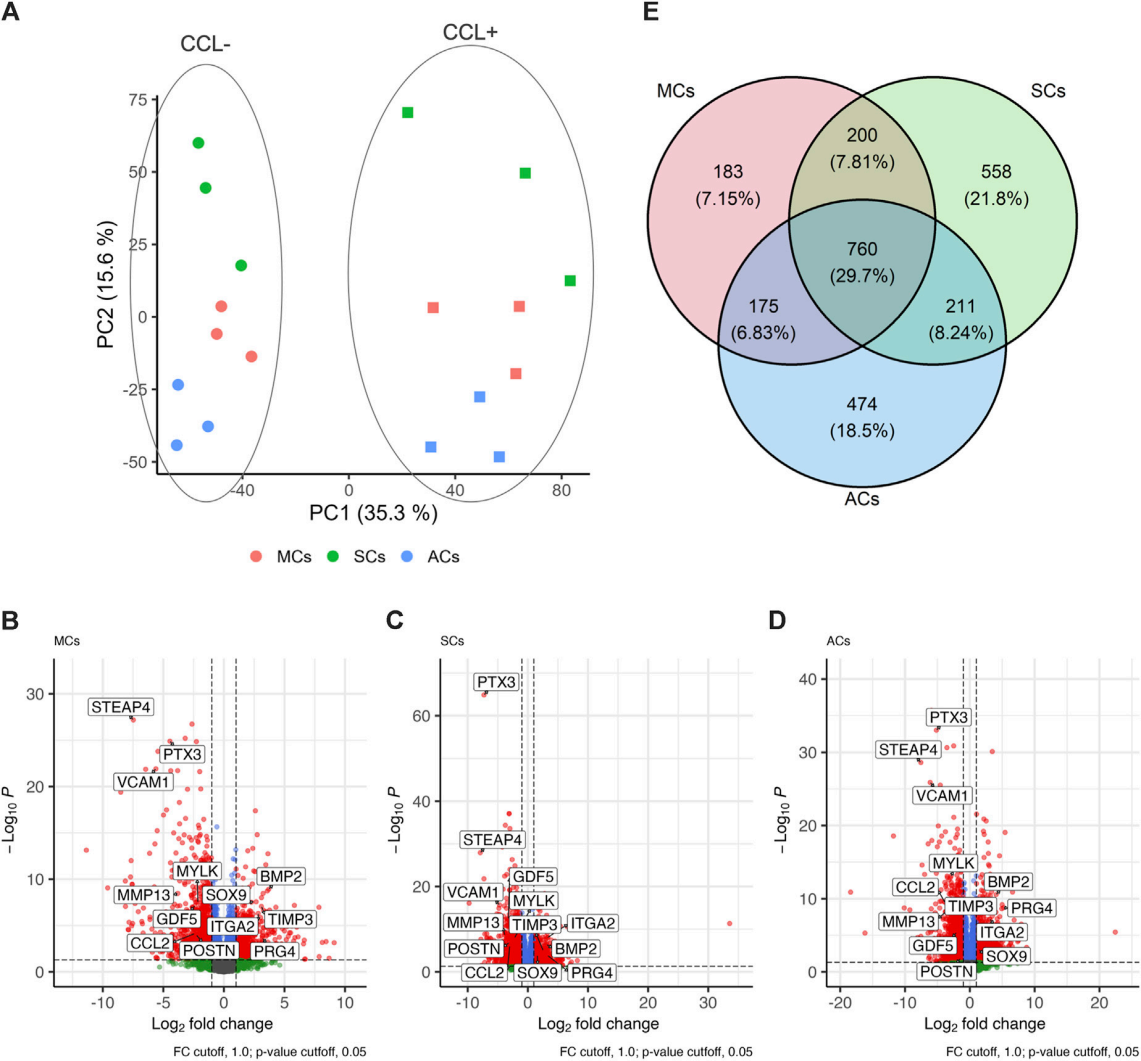
Transcriptome analysis of 3D-cultured meniscus-derived cells (MCs), synovium-derived cells (SCs), and articular cartilage-derived cells (ACs) with or without cyclic compressive loading (CCL) stimulation **(A)** Normalized read count-based principal-component analysis (PCA, *n* = 3 donors). The circles and squares represent CCL− and + samples, respectively. **(B–D)** Volcano plot highlighting the differential gene expression analysis between CCL− and + samples in MCs, SCs, and ACs. Each data point corresponds to a gene, plotted based on its log2 fold change (*x*-axis) and adjusted *p*-value (*y*-axis). Genes with statistically significant differential expression (adjusted *p*-value <0.05) are highlighted in red. The vertical and horizontal dashed lines indicate the threshold for log2 fold change (±1) and significance threshold, respectively (adjusted *p*-value = 0.05). **(E)** Venn Diagram illustrating the differentially expressed genes (DEG) overlap among MCs, SCs, and ACs upon CCL. The numbers in each region represent unique and shared DEG counts. The intersections display genes that are commonly regulated across the 3 cell types upon CCL stimulation. The percentages were calculated based on the total DEG number for each cell type.

Next, we conducted RNA-seq analysis to investigate how CCL could affect the transcriptomic changes in 3D-cultured MCs, SCs, and ACs. Our sequencing and subsequent data processing yielded high-quality RNA-seq data containing 16,520 genes per sample. Our principal-component analysis (PCA) demonstrated distinct experimental group clustering by loading (CCL^+/−^) and cell type (Figure 3A). CCL exposure elicited significant transcriptome changes in MCs, resulting in 1,318 differentially expressed genes (DEGs; |log2 fold change| > 1 and adjusted *p*-value <0.05), comprising 430 and 888 up- and downregulated genes, respectively (Figure 3B). SCs subjected to CCL exhibited 1,729 DEGs, including 645 and 1,084 up- and downregulated genes, respectively (Figure 3C). Similarly, CCL-treated ACs displayed 1,620 DEGs, encompassing 578 and 1,042 up- and downregulated genes, respectively (Figure 3D). MCs, SCs, and ACs displayed 760 (29.7%), 183 (7.2%), 558 (21.8%), and 474 (18.5%) overlapping, MC-, SC-, and AC-specific DEGs, respectively. (Figure 3E).

### Mechanoreceptor and cartilage-related gene expression in response to CCL

**FIGURE 4:**
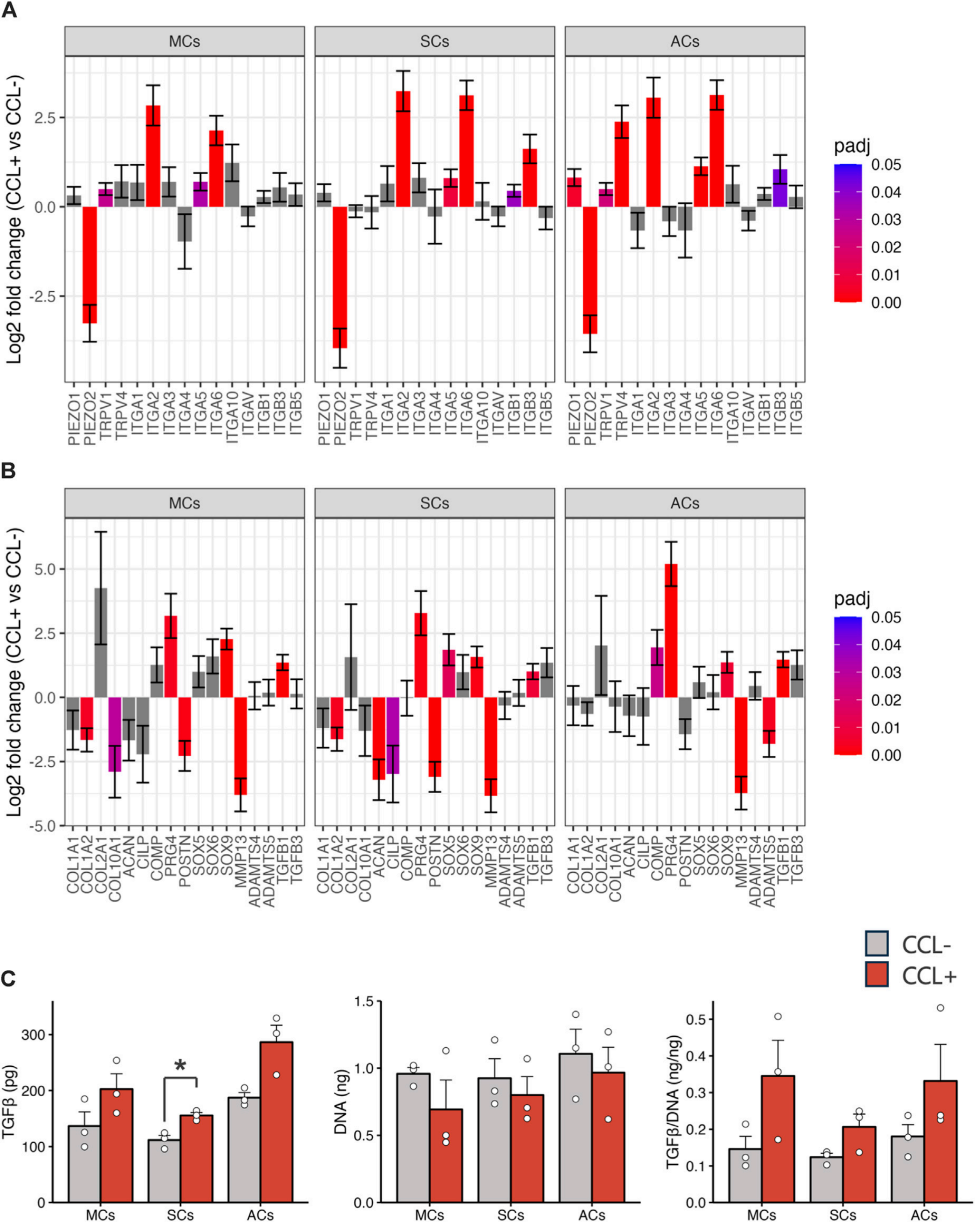
Chondrocyte-related gene expression and TGF-β1 protein concentration **(A, B)** Mechanoreceptor- **(A)** and chondrocyte-related **(B)** gene expression analysis based on the RNA-seq results. The vertical axis and error bars represent the log2 fold change and its standard error (SE), respectively. The bar colors denote adjusted *p*-values. **(C)** TGF-β1 protein concentrations in the culture supernatants and DNA content in the 3D-cultured cells on day 14. Light gray and red bars represent samples without and with cyclic compressive loading (CCL) stimulation, respectively. n = 3 donors. mean (SEM). *p < 0.05, by Welch’s *t*-test.

Subsequently, we investigated mechanoreceptor- and cartilage development-related gene expression via RNA-seq analysis. Our data revealed significant expression changes in representative mechanosensory receptors, including upregulated integrin family members (specifically ITGA2, ITGA5, and ITGA6) upon CCL stimulation across all cell types (Figure 4A). Furthermore, within the representative cartilage-related gene list, we observed notable alterations (i.e., PRG4, TGFB1, and SOX9 upregulation as well as MMP13 and POSTN downregulation) (Figure 4B). Upon CCL stimulation, the TGF-β1 protein concentration in the supernatant of 3D-cultured SCs increased significantly (*p* = 0.017), with a trend towards an increase also observed in MCs and ACs (*p* = 0.15 and *p* = 0.072, respectively). (Figure 4C).

### CCL-mediated meniscal cell differentiation transcriptomic regulation

**FIGURE 5:**
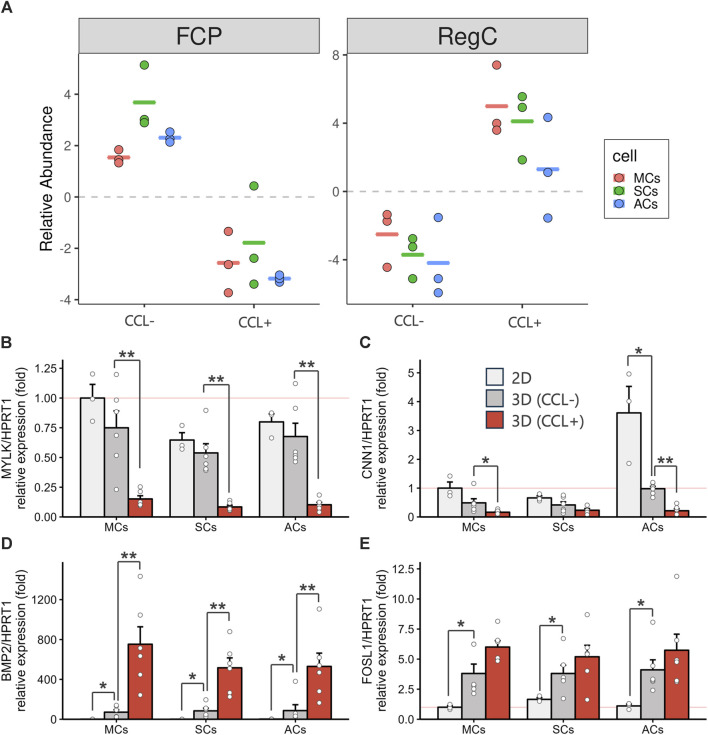
3D-cultured meniscus-derived cells (MCs), synovium-derived cells (SCs), and articular cartilage-derived cells (ACs) differentiation assessment into the mature meniscal cell with or without cyclic compressive loading (CCL) **(A)** Relative fibrochondrocyte progenitors (FCPs) and regulatory chondrocytes (Reg Cs) cell proportion estimations using BisqueRNA. Each cell type population in the 3D scaffold was estimated from bulk RNA-seq transcriptome data (n = 3 donors) and meniscus single-cell RNA-seq public data. **(B–E)** Gene expression of MYLK **(B)**, CNN1 **(C)**, BMP2 **(D)**, and FOSL1 **(E)** as measured by RT-qPCR. Expression level were normalized to HPRT1 and were presented as relative expression with MCs 2D set as the baseline (value of 1). *n* = 6 donors. mean (SEM). *p < 0.05, **p < 0.01, by Wilcox rank sum test.

The previous single-cell RNA-seq study identified cell populations and lineages within the human meniscus, highlighting that MYLK- and CNN1-expressing fibrochondrocyte progenitors (FCPs) represented undifferentiated cells. In contrast, BMP2- and FOSL1-expressing regulatory chondrocytes (Reg C) and ADAMTS4- and MMP1-expressing fibrochondrocytes (FC) denoted more mature and differentiated cell types (Sun et al., 2019). Using the transcriptome information from the previous single-cell RNA-seq and the current bulk RNA-seq datasets, we investigated how CCL could influence meniscal differentiation in 3D-cultured cells. We performed a cell type deconvolution analysis, revealing reduced estimated FCP and increased Reg C populations upon CCL stimulation (Figure 5A). In addition, we conducted RT-qPCR analyses for FCP and Reg C marker gene expression evaluation. CCL stimulation significantly downregulated MYLK (*p* = 0.0043 in MCs and *p* = 0.0022 in ACs) and CNN1 (*p* = 0.015 in MCs and *p* = 0.0022 in ACs) expression among 3D-cultured MCs and ACs as well as that of MYLK (*p* = 0.0022) in SCs (Figures 5B, C). In contrast, CCL stimulation induced a marked BMP2 (*p* = 0.0022 in MCs, *p* = 0.0022 in SCs, and *p* = 0.0087 in ACs) and FOSL1 (*p* = 0.065 in MCs, *p* = 0.31 in SCs, and *p* = 0.40 in ACs) expression increase across all 3D-cultured MCs, SCs, and ACs (Figures 5D, E). These results indicate that CCL stimulation orchestrates a transcriptional shift from progenitors toward a more mature meniscal cell phenotype.

### Gene Ontology and KEGG pathway enrichment analysis

**FIGURE 6:**
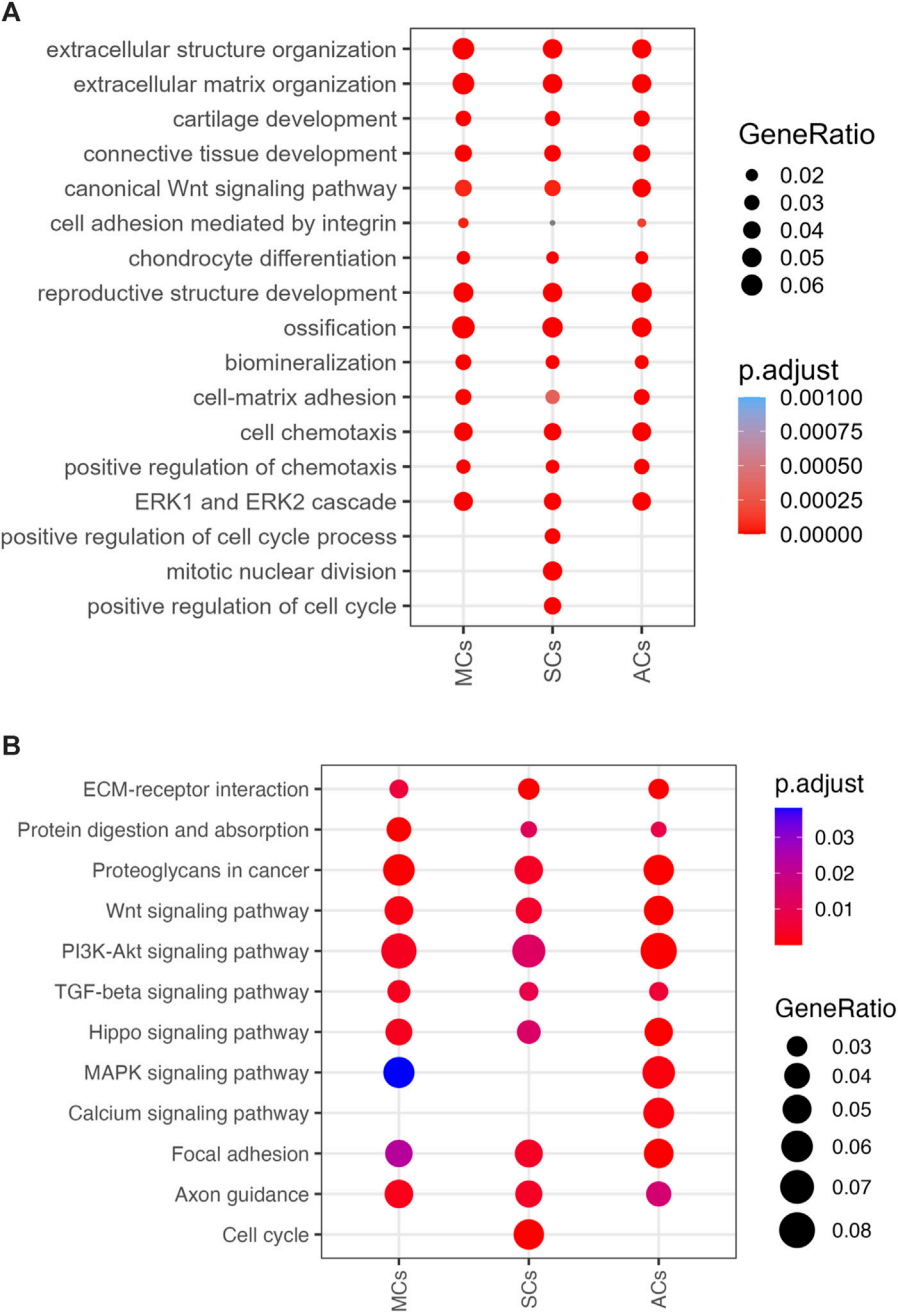
3D-cultured cell enrichment analysis and mechanical properties **(A)** The top enriched Gene Ontology Biological Processes (GOBP) terms for differentially expressed genes (DEGs) upon cyclic compressive loading (CCL) stimulation. The x- and *y*-axes represent the cell types and GOBP terms, respectively. Circle sizes and colors correspond to the GeneRatio and significance level, respectively (adjusted *p*-value). **(B)** Enriched KEGG pathways among the DEGs upon CCL stimulation. The x- and *y*-axes represents the cell types and KEGG pathways, respectively. Circle sizes and colors indicate the GeneRatio and significance level, respectively (adjusted *p*-value).

To further investigate DEG functions and interconnections, we performed Gene Ontology (GO) and Kyoto Encyclopedia of Genes and Genomes (KEGG) pathway analyses. Our GO enrichment analysis provided a comprehensive overview of the biological processes associated with the DEGs in MCs, SCs, and ACs under CCL. This analysis revealed a wide range of processes, notably including extracellular matrix (ECM) organization, cartilage development, inflammation response, angiogenesis, and cell migration. (Figure 6A). Following up on the insights gained from the GO analysis, our KEGG pathway analysis further delineated the specific signaling pathways that these DEGs are involved in. Notably, pathways such as ECM-receptor interaction, TGF-beta signaling, PI3K-Akt signaling, and NF-kappa B signaling were identified as being enriched. (Figure 6B). These analyses suggest a complex mechanism by which MSCs respond to compressive stress, through a coordinated activation of biological processes and signaling pathways essential for meniscal regeneration.

## Discussion

As a significant observation, our study revealed that CCL promotes the differentiation of MSCs derived from meniscus, synovium, and articular cartilage within ACMS, particularly towards a Reg C phenotype marked by upregulation of BMP2 and FOSL1 as key mature meniscal cell markers.

In mechanobiology study, various mechanical loading methods are employed, including tensile stress, static load, cyclic or dynamic compressive loading, and hydrostatic pressure. For this experiment, CCL at 40 kPa of load and at frequency of 0.5 Hz was chosen to simulate the compressive loading on the inner meniscal cells during “slow walking activity”, considering rehabilitation scenarios post-meniscal injury. While accurately reflecting the physiological pressures exerted on human menisci was challenging, the magnitude of the load was set based on the study that showed the meniscus experiences compressive loading of 20–80 kPa *ex vivo* (Richards et al., 2005) and our previous studies (Shimomura et al., 2014; Kanamoto et al., 2021). The choice of 0.5 Hz mimicking “slow walk” was informed by the recent findings (Sun et al., 2023). We selected a 7-day CCL period to investigate beyond the initial, predominantly inflammatory responses observed with only 1 day of loading in our previous research (Shimomura et al., 2014). Such acute inflammatory reactions are critical but are just the onset of a sequence of healing events. Over the course of a week, the cellular activity transitions from this inflammatory phase to processes more indicative of the beginning of reparative mechanisms, such as ECM remodeling (Petri et al., 2012; Zellner et al., 2015). Moreover, the choice of a 7-day loading protocol mirrors clinically relevant time points, providing insights into the cellular and molecular events that occur in the early phase post-injury. This information is crucial for informing clinical decisions on timing and types of interventions. Our findings from the 40-kPa of CCL at frequency of 0.5 Hz for 7 days provide a foundation for understanding the early cellular events in meniscal repair, but they also underscore the necessity for long-term studies to fully capture the spectrum of tissue adaptation and repair over time.

Our transcriptomic analysis identified several genes (e.g., TGFB1, SOX9, PRG4, and POSTN) involved in cartilage homeostasis and development, as DEGs in response to CCL. In particular, PRG4 (i.e., lubricin, produced by cells in the superficial zone of the synovium, articular cartilage, and meniscus) upregulation significantly influences chondrocyte function and cartilage homeostasis (Jay and Waller, 2014; Takahata et al., 2022). PRG4-expressing cells can act as progenitors for articular cartilage repair, suggesting a potential for contributing to cartilage repair, including possibly the meniscus (Massengale et al., 2023). However, PRG4’s lubricative function can reduce friction, potentially preventing adhesions during the healing phase. The comprehensive impact of PRG4 upregulation on meniscal regeneration remains an area requiring further investigation. Moreover, our GO enrichment analysis revealed a distinct enrichment of GO biological process terms associated with cartilage development and chondrocyte differentiation. On the contrary, our findings showed that CCL did not increase the expression of COL1A1 and COL1A2, which are markers typically found in a variety of connective tissues, including meniscus. This suggests that CCL may not significantly promote differentiation towards a fibroblast-like phenotype. Given these findings, it can be concluded that CCL is likely to support the enhancement of fibrochondrocyte-like properties, characteristic of the inner meniscus, rather than fostering fibroblast-like traits of the outer meniscus.

Since meniscus phenotype-specific gene expression had not yet been characterized, the conventional meniscal differentiation assessment approach predominantly depended on chondrocyte marker (e.g., SOX9, COL2A1, and ACAN) expression (Gunja et al., 2009; Zellner et al., 2017). Nonetheless, emerging number of studies advocate that meniscal and articular cartilage tissue exhibit distinct phenotypes, in spite of their often cited similarities (Son and Levenston, 2012). A recent single-cell RNA sequencing study identified multiple subpopulations within the human meniscus and shed light on their differentiation trajectory. Remarkably, FCPs differentiated into Reg Cs (Sun et al., 2019). In this study, we adopted the genes described in the aforementioned previous study as meniscal differentiation markers. Our RT-qPCR analyses revealed that the MC, SC, and AC transcriptional profiles in three-dimensional mechanobiological culture systems shifted from FCP-like toward Reg C-reminiscent ones, epitomizing a mature cell population within the meniscus. Furthermore, our RNA-seq analysis, complemented by the GO enrichment and KEGG pathway analyses, proved that CCL stimulates cell differentiation and ECM metabolism. Taken together, our results suggest that our 3D biomechanical culture system promotes transcriptional differentiation toward phenotypes that more closely approximate mature meniscal cells.

The underlying mechanisms of mechanical signal transformation into biological changes in the meniscus remain unclear. However, integrins are confirmed mechano-transduction mediator candidates with a well-documented ability to transmit mechanical force signals from the ECM to the intracellular environment (Loeser, 2014). Kanamoto *et al.* reported that integrin α2β1 played a significant role in CCL mechanotransduction in articular cartilage-derived cells (Kanamoto et al., 2021). Furthermore, Zhang *et al.* demonstrated that cyclic hydrostatic compressive force enhanced meniscal fibrochondrocyte proliferation via integrin α5β1 (Zhang et al., 2019a). In accordance with these results, our study revealed significant ITGA2, ITGA5, and ITGA6 gene upregulation in response to CCL stimulation. Notably, our KEGG pathway analysis unveiled a significant enrichment in ECM-receptor interaction- and Focal Adhesion-related pathways, suggesting that CCL-mediated responses might be triggered through integrin-mediated cellular interactions with the extracellular matrix.

Numerous investigations have been conducted to determine optimal MSC tissue sources for meniscal repair and regeneration without reaching a definitive consensus. In our study, we investigated differentiation capacity-related differences among MSCs derived from three tissues and observed no significant disparities. Despite the lack of marked differences, our RNA-seq analysis hinted at subtle transcriptional distinctions in synovium-derived MSCs upon mechanical stress, suggesting that overarching characteristics might be similar, though nuanced variations in different tissue source-derived MSC molecular behavior could exist. Importantly, MSC-related studies are susceptible to a range of factors, including cell extraction methods, culture protocols, and differentiation conditions, exemplified by a study describing a higher chondrogenic differentiation capacity in meniscus-derived MSCs compared to bone marrow-derived MSCs, whereas another study presented opposing results (Shen et al., 2014; Huang et al., 2016; Zellner et al., 2017). Further investigations would be required to explore the optimal MSC sources for meniscus regeneration.

This study also has certain limitations. First, the 3D culture conditions such as MSC passage, mechanical stress types, and additional growth factors were fixed, potentially affecting the MSC transcriptomic response to CCL. Second, the MSCs we used derived from the tissue of patients with osteoarthritis, its pathological environment potentially influencing inherent MSC characteristics. Third, the long-term impact of the transcriptional changes toward meniscal differentiation on the mechanical properties of scaffold requires further investigation. Despite these limitations, to the best of our knowledge, this study is the first to evaluate the effects of CCL on MSC differentiation within ACMS towards Reg C phenotype using specific markers.

In conclusion, our results shed light on the potential of mechanical stress to facilitate the transcriptional differentiation of MSCs derived from the meniscus, synovium, and articular cartilage into mature meniscal cells, specifically Reg C phenotype.

## Data Availability

The data presented in the study are deposited in the National Center for Biotechnology Information Gene Expression Omnibus (GEO) repository, accession number GSE 256243.
